# Leadership in Integrated Care Networks: A Literature Review and Opportunities for Future Research

**DOI:** 10.5334/ijic.5420

**Published:** 2020-08-11

**Authors:** Matthias Mitterlechner

**Affiliations:** 1 University of St. Gallen, St. Gallen, CH

**Keywords:** integrated care, network, leadership

## Abstract

**Introduction:**

In many countries, elderly patients with chronic conditions require a web of services delivered by several providers collaborating in inter-organisational networks. In view of their global importance, it is surprising how little we know how these networks are led. Like traditional organisations, networks require leadership to function effectively. This paper reviews central characteristics of leadership in integrated care networks and proposes opportunities for future research.

**Theory and methods:**

Analysing 73 studies published in leading academic journals, this paper consolidates research on leadership media, practices, activities and outcomes, covering the network, policy and organisation levels of analysis.

**Results:**

Findings indicate that the field has focused on leadership media and outcomes at the network level. They also suggest that leadership in integrated care networks faces multiple tensions. Future research could usefully provide a fuller picture by examining leadership practices, activities and outcomes at the policy and organisation level, integrating advances in the wider leadership literature.

**Discussion and conclusion:**

These findings contribute to the debate on leadership in integrated care networks. They also inform practice, drawing attention to persistent tensions as a core leadership challenge and offering latest scholarly evidence practitioners can use to reflect on and advance their own leadership practice.

## Introduction

This article reviews research on leadership in integrated care networks. It is timely as scholars and policy makers regard integrated care as a key part in reforming healthcare systems to cope with demographic aging, the rising prevalence of chronic diseases and the growing demand for long-term care [1,1]. In this context, integrated care is seen as a promising approach for enhancing the efficiency of service delivery and for improving patients’ and populations’ care experiences and health outcomes [2]. Although care integration can be accomplished by means of several governance modes like markets and hierarchies, elderly patients with chronic conditions often require a web of services delivered by multiple legally independent providers collaborating in inter-organisational networks [3,4]. This pertains particularly to patients with complex and unpredictable needs [1,5]. The list of countries advancing network-based care integration is long. It includes, among others, Australia [6], Canada [7], Denmark [8], Germany [9], the Netherlands [10], Switzerland [11], Taiwan [12], the UK [13] and the US [14].

In view of their worldwide importance, it is surprising how little we know about how integrated care networks are led. Amelung et al. observe that “Leadership is certainly one of the neglected topics in integrated care” [15, p. 221]. This gap in the literature is unfortunate for several reasons. First, leadership can make a difference. As Goodwin underlines, one of the core challenges for the successful adoption of integrated care systems is leadership [16]. Second, the leadership challenge is greater in inter-organisational networks than in traditional organisations due to a lack of hierarchical influence. In networks, leadership involves the coordination of several legally autonomous organisations [17]. Moreover, research shows that successful networks are not static but evolve dynamically, which requires ongoing leadership attention over time [18]. Third, the leadership challenge is exacerbated by the way health and social care services are provided. In these sectors, service provision involves the coordination of activities among multiple, highly specialised expert organisations operating at different levels of the system [15].

An explanation for this apparent knowledge gap may be that previous researchers have used different terminologies, methods, levels of analysis and theories in their work. For example, the topic can be studied from network theory, integrated care theory or other theoretical angles, each perspective using its own definitions and illuminating different aspects of the phenomenon. Against this background, this paper aims to review central characteristics of leadership in integrated care networks and outline avenues for future research. It thereby adds to the literature on integrated care and, more narrowly, the debate on leadership in integrated care networks. To this end, it proceeds as follows. The next two sections define key terms, delineate conceptual boundaries and describe the methods used for conducting this review. The third section summarises our current knowledge on leadership in integrated care networks. On this basis, the paper suggests opportunities for future research and closes with summarising considerations.

## Theoretical background

The three key terms used in this paper – integrated care, networks, and leadership – are all “polymorphous” concepts, which have been defined from various theoretical and disciplinary angles and with multiple objectives [1, p. 5]. To establish common understanding, this section defines the three terms and specifies the conceptual boundaries guiding this study.

First, the concept of *integrated care* has been applied in various ways and from different professional and disciplinary perspectives including public health, public administration, management and psychology [1]. While all of these perspectives are legitimate, this study follows Kodner and Spreeuwenberg, who define integrated care as “a coherent set of methods and models on the funding, administrative, organisational, service delivery and clinical levels designed to create connectivity, alignment and collaboration within and between the cure and care sectors. The goal of these methods and models is to enhance quality of care and quality of life, consumer satisfaction and system efficiency for patients with complex, long-term problems cutting across multiple services, providers and settings” [adapted from 19, p. 3]. This definition suits to this study because it addresses multiple levels of analysis, outcomes of care and inter-organisational collaboration.

Second, similar to integrated care, research on *inter-organisational networks* is highly fragmented. Researchers have applied the term “network” both as an analytic perspective and as a concept for describing a separate mode of governing economic activities [20]. On the one hand, defining networks as sets of nodes and ties, social network analysts have studied the antecedents and consequences of networks at different levels, including the inter-organisational level [21]. On the other hand, researchers in the governance tradition conceive of inter-organisational networks as a distinct mode of governing economic exchange situated between markets and hierarchies. In networks, organisations coordinate activities through reciprocal, preferential and mutually supportive actions rather than through discrete market exchanges or by administrative fiat [3]. This study is situated in the governance tradition and builds on Müller-Seitz and Sydow [17], who define an inter-organisational network as “a social system in which the activities of at least three formally independent legal entities are coordinated in time-space, i.e., there is some reflexively agreed upon inter-firm division of labour and cooperation among the network members” (p. 108). This definition has several implications. First, it excludes dyads, recognising that third actors give such relationships a distinct social quality, e.g. one actor’s option to play two or more others against each other for his or her own benefit [22,23]. Second, it requires that actors are aware of one another to connect, align and coordinate activities, excluding loosely structured collections of organisations [23]. Third, it is open to several types of integrated care networks like cancer, diabetes, youth care or HIV networks. Fourth, it includes multiple directions and covers vertical, horizontal, cross-sectoral or population-centred networks [1]. Finally, while focusing on the network level, it recognises that networks are recursively situated in “neighbouring” levels including those of the institutional field (policy level) and network members (organisation level) [23].

Third, the notion of *leadership* has long attracted significant interest among management researchers and social scientists more broadly. Most research has been leader-oriented, studying the traits, abilities and actions of effective leaders [24]. More recently, researchers have made calls to pay greater attention to how leadership evolves in concrete social contexts like inter-organisational networks, and to study the interaction between these contexts and leaders’ activities [25,26]. Considering these calls, this literature review builds on Huxham and Vangen [27,28], who define network leadership as being concerned with the “mechanisms that ‘make things happen’” [27, p. 415]. They suggest that these mechanisms include leadership media and leadership activities. *Leadership media* refer to contextual structures and processes (formal and informal communication instruments) through which network agendas are created and implemented. In many cases, leadership media are beyond the direct control of network members as they are imposed by external actors or emerge from previous leadership activities as unintended outcomes. *Leadership activities* refer to what actors, i.e. network member organisations and third parties, do to move a network forward. Generic examples are managing power, representing and mobilising network member organisations or empowering those who can deliver collaboration aims [28]. Enabled and constrained by leadership media, leadership activities are often imbued with tensions in the sense of persistent contradictions between opposite elements so that actors’ outcomes are not always as intended [23,27,28,29]. In contrast to other leadership theories, this definition is less interested in the difference between leadership and management [15,30]. It also reaches beyond leader-oriented theories emphasising individual actors’ traits, styles, behaviours or transformational skills [24]. Avoiding an overly heroic image of leadership in light of the above mentioned complexities of integrated care networks, it recognises how leadership activities are enabled and constrained by contextual structures and processes [31].

## Review methodology

The methodology used in this paper is informed by guidelines for the conduct of reviews [32,33,34,35] and similar review articles [36,37]. To ensure reliability, all steps were performed independently by the author and a research associate. The two separate analyses produced a few minor differences that were jointly resolved by double checking the data. The review started with the access to scientific databases. The search was restricted to peer-reviewed articles in English-language journals. Although this focus excludes non-English articles, contributions in edited books, and monographs, it enables transparency and provides insights into the most important aspects of the scholarly debate on the topic [32,36,37,38]. The date of publication was open up to June 2019. The databases included the Cochrane Library, EBSCOhost, MeSH terms, PubMed, and the Web of Science. Synonyms for the terms leadership, networks and integrated care were matched. While a broad range of synonyms was used to include as many studies as possible, the search was narrowed with the term “inter-organisational” (and related synonyms) to exclude inter-personal, intra-organisational and other forms of networks. Appendix 1 provides an overview of the search strings used.

**Figure 1:**
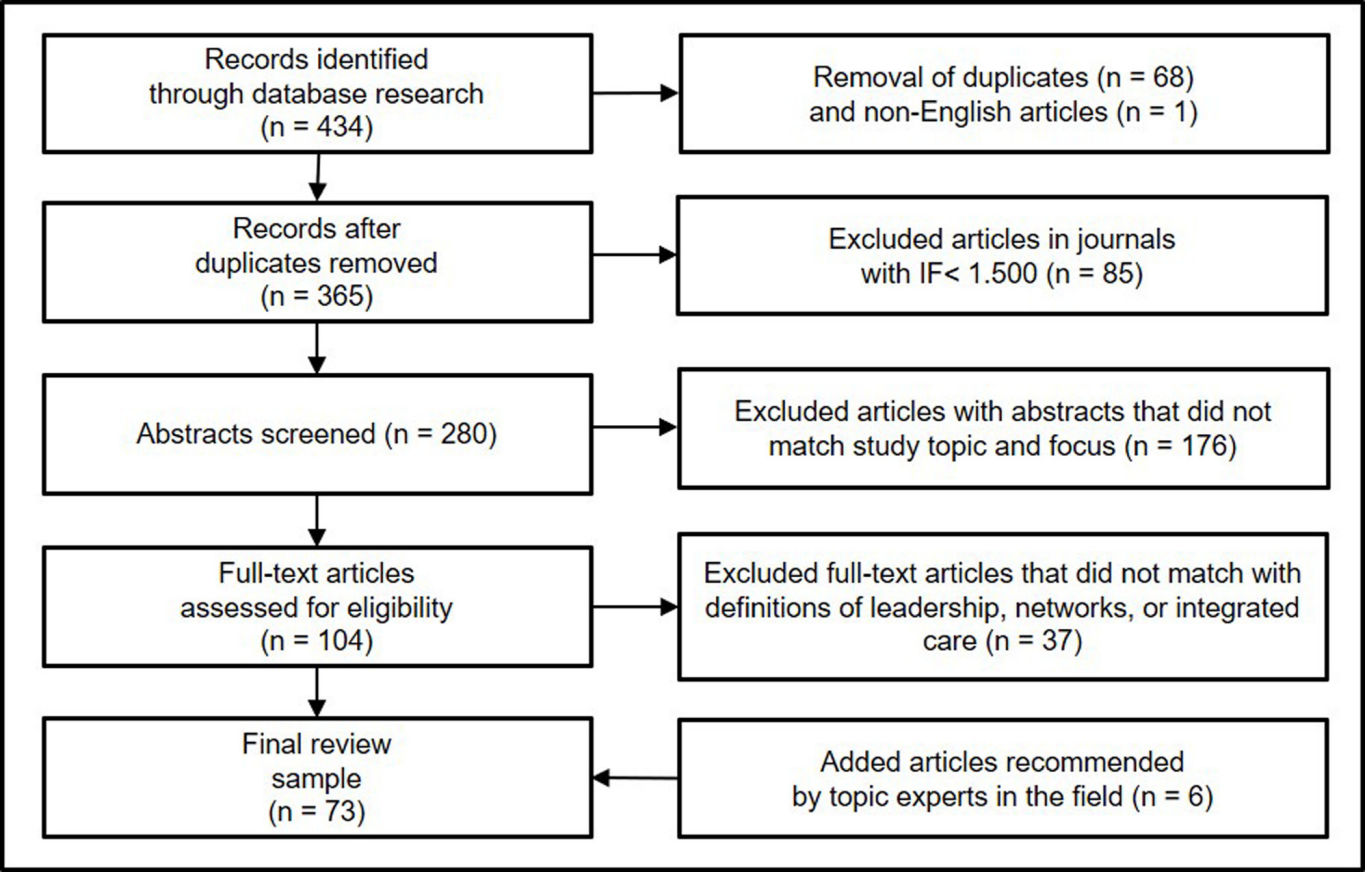
Sampling approach.

After the removal of 68 duplicates and 1 non-English article, the search produced 365 hits (see Figure 1). To improve the quality of the sample, only articles that were published in top journals with a 5-year impact factor of 1.500 or more (as of June 2019) were included, which reduced the number of relevant articles to 280. While this focus on impact factors has limitations, it provides access to state-of-the-art and peer-reviewed knowledge and has become common practice in similar reviews [e.g. 39,40]. To identify relevant studies about leadership in integrated care networks, the abstracts of these articles were reviewed and 176 articles that did not relate to the topic were excluded.

The full texts of the 104 remaining articles were reviewed with regards to how they corresponded to the definitions of leadership in integrated care networks as outlined above. Considering these criteria, 37 articles were excluded. Using this approach, 67 journal articles corresponding to the definition of leadership in integrated care networks were identified. Furthermore, 6 articles recommended by topic experts in the field were added. These 73 articles constitute the core sample of this review.

In a next step, the 73 articles were analysed and coded according to 11 different criteria, which were derived both deductively (e.g. by drawing on the definitions of integrated care, networks, and leadership as outlined above) and inductively (e.g. by reading the articles in depth and refining the focus of the review). The findings of the analysis are described in the following section and summarised in Appendix 2.

## Findings

The findings show that leadership in integrated care networks is a *relatively young* field. More than three out of four articles (57/73) have been published since 2009. The top three *journals* publishing articles on the topic are the International Journal of Integrated Care (7), Health Policy (5) and Health Care Management Review (4). To study the topic, researchers use various *theoretical lenses*. The most important theories are network theory (15), integrated care theory (11), social network theory (7), organisation theory (6) and leadership theory (4), whereby some papers combine two or more lenses. In terms of *methods*, most studies are qualitative (41). Fewer studies use quantitative (17), mixed (11) or non-empirical conceptual (4) methods. Sample sizes in empirical papers range from single case studies to 104 networks. Most studies are conducted in the US (23), followed by the UK (12), the Netherlands (9), Canada (9) and Australia (5).

Reviewing the current body of research on leadership in integrated care networks, this paper draws on Huxham and Vangen [27,28], who suggest that leadership in networks includes leadership media and leadership activities. In the review, it was found that a few studies analyse leadership media and activities in their interplay. To consider these articles, a third category called “leadership practices” was developed, a term signifying a recursive relationship between social structure and action [31]. In addition, the paper acknowledges that integrated care networks are not isolated systems but situated in a policy and member organisation level [23]. Table 1 shows the resulting analytical framework and findings. Articles covering several leadership mechanisms and/or levels of analysis appear in more than one cell of the table [e.g. 41].

**Table 1:** A map of the field of leadership in integrated care networks (including double counts).

	Leadership Media (58 studies)	Leadership Practices (14 studies)	Leadership Activities (23 studies)

Policy level (30 studies)	Public governance [6,9,41,42,43,44] Legislation/regulation [7,44,45,46,47] Funding [11,14,41,48,49,50,51,52,53] Performance control [48,54,55] Social environment [56,57]	Continuous learning [58]	Exertion of influence by governments and health departments [59,60,61] Using dialogue vs. the shadow of hierarchy [62]
Network level (50 studies)	Network governance [11,45,49,63,64,65,66,67,68,69] Network trust [10,12,41,48,70,71,72,73] Network structures [63,74,75,76] Network geography [50,67,77]	Creating connectivity/consensus [78] Co-creation [79] Planning [80] Managing performance [81,82] Continuous learning [83,84] Sharing information [85] Pooling resources/expertise [13,86,87]	Committed/credible leaders [88,89,90,91,92,93] Nurturing linkages [41,51,94,95,96,97] Aligning goals and creating shared understandings [95,98]
Organisation level (15 studies)	Professional traditions/work practices [46,48,50,53,99,100] Organisational priorities and resources [43,45]	Multilateral boundary spanning [101] Hospital ownership [102]	Leaders’ motivation [8,44,103,104] Leaders’ tenure [66]

### Leadership media

Leadership media refer to the structures and processes making things happen in an integrated care network [27,28]. At the *network level*, many studies emphasise the role of *network governance* [105], i.e. structures and processes of authority and control to coordinate network activities [11,45,49,63,64,65,66,67,68,69]. Regarding governance structures, some authors suggest that effective networks rely on centralised governance, in which network activities are coordinated through a single network member [67]. Grusky, for example, suggests that powerful lead agencies are more likely to persuade other network members to give up some of their autonomy and engage in network-based care coordination [66]. Others point to potential drawbacks of this governance mode, showing how it may lower network members’ citizenship and behavioural commitment [63]. They tend to argue for shared governance in which activities are coordinated by all network members [45]. Developing these arguments, some authors find that there are several paths to success. Given sufficient public funding, activity coordination is enabled by centralised governance combined with a strong exercise of leadership activities (see next section) or shared governance combined with adequate governance processes [11]. Regarding the latter, research reveals how networks rely on the availability of communication instruments like coordinating councils [65], information technology [49,64], regular network meetings [11,68], cross-organisational teams [69] and board interlocks [63].

Besides governance structures and processes, studies examine the role of *trust* among network members [10,12,41,48,70,71,72,73]. They show how trust mediates network members’ willingness to collaborate in care planning [41,72] and exchange patient-related information through electronic medical records [12]. Trust is a prerequisite for network members to express uncertainty [10] and share professional knowledge [71,73]. It can easily erode through recognition asymmetries among network members of each other’s skills and differences in culture and attitudes towards change [70]. It can also erode through capacity and financial imbalances within a network, which may raise doubt that all network members act fairly to create positive outcomes for the good of the whole [48,70].

Analysts also study the impact of *network structures* [63,74,75,76], arguing that networks rely on network efficiency and density [63,75,76]. In contrast, network breadth can decrease performance, creating difficulties in reaching consensus and creating trust within a network [75]. Moreover, performance is improved by network members working through cliques, which unite complementary services and establish trust among clique members [74].

Finally, several authors suggest an association between integrated care networks and *geography* [50,67,77]. Some argue that activity coordination is facilitated by proximity among provider organisations [67,77]. Others question this positive relationship and suggest that geographical co-location does not automatically lead to inter-organisational collaboration, pointing out that collaboration is additionally mediated by structures and processes at the policy and organisation level of analysis [50], findings reviewed next.

At the *policy level*, the importance of *public governance structures*, i.e. context-specific mixtures between hierarchies, markets and networks [106], is subject to considerable debate [6,9,41,42,43,44]. Some writers note that pro-competitive policy reforms increase inter-organisational collaboration, e.g. in the areas of sharing medical specialists [42] or adopting joint data sharing standards [44]. Others, by contrast, find that market-based competition undermines collaboration, observing how it complicates the formation of disease management [41], quality improvement [43] and comprehensive primary care networks [6]. Placed between these views, Bode et al. argue that networks seem to struggle with tensions resulting from conflicting public governance regimes, whereby these tensions are context-specific and tied to the mix of public governance in each place [9].

Moreover, several studies provide insights into how integrated care networks are enabled and constrained by *government legislation* [7,44,45,46,47]. Some show how government reforms aimed at improving collaboration among providers support the creation of horizontal and vertical service networks [7] and exchange of patient-related information [44,47]. Others describe how integrated care networks are constrained by the absence of supportive legislation [45] or legislation contradicting the development of locally useful solutions [46].

Legislation has a particular impact by *providing funding and creating financial incentives* [11,14,41,48,49,50,51,52,53]. On the one hand, studies suggest that effective networks depend on sufficient public funds [11,53], which can be used to incentivise networks with coordination fees or kick-start collaboration [51,52]. On the other hand, they show how financial incentives can undermine collaboration [50]. Several studies recommend coordinating different funding streams and creating collaborative financial contracts among providers and insurers, which support patient coordination and information exchange across organisations [14,41,48,49].

Research also sheds light on the enabling and constraining effects of public *performance control* structures, illuminating how networks are constrained by backward looking structures that reward the success of single organisations [48,54,55]. From the perspective of each network member, these structures focus priorities internally rather than on relationships with other organisations [48,55]. Complementing these structures with forward looking and network-oriented performance control may facilitate activity coordination among different actors involved in the policy-making process [54].

Finally, writers note that integrated care networks are affected by the broader *social environment* in which they are embedded [56,57]. Some find that networks are more likely to emerge in communities with higher levels of local social capital, measured by active participation in public life, trust and voter participation [56]. Others, however, caution that even in communities with high levels of social capital, organisations are less like to engage in networks if they do not trust one another or have divergent organisational agendas [57].

At the *organisation level*, the literature emphasises that integrated care networks are enabled and constrained by network members’ *professional work practices* [46,48,50,53,99,100]. Network-based collaboration arises more easily if it does not disturb professionals’ previous work routines [46,100]. By contrast, it is complicated by network members’ divergent treatment approaches [50], HR practices [99] and quality assurance practices [48]. Against this background, the literature argues that the formation of integrated care networks needs to be accompanied by considerable investment in supporting the change of professional work practices within network member organisations [48,53]. Moreover, it draws attention to the mediating role of *organisational priorities and resources* [43,45]. Integrated care networks are enabled and constrained by competing priorities [43] and capacity and resource limitations within network member organisations [45].

### Leadership activities

Leadership activities refer to what network members and third parties do to move a network forward [27,28]. At the *network level*, research addresses the activities of network leaders [88,89,90,91,92,93]. It shows that integrated care networks depend on credible and committed “network champions” who promote collaboration to other network members and stakeholders at the policy level [90,91,92,93]. These leaders exert influence by encouraging communication among stakeholders and by delivering strong messages regarding the importance of collaboration [88,89,90]. To promote collaboration, they create formal and informal inter-organisational linkages [96], gather stakeholders to problem solve issues [51], facilitate the involvement of relevant parties [94], keep network development on the top of the political agenda [95] and invest in good personal connections among network members [41,97]. Moreover, they use local events to articulate network goals [98] and create shared understanding of network values [95].

At the *policy level*, research is concerned with the activities of governments and health departments in advancing integrated care networks [59,60,61,62]. These actors are able to exert influence because they are authorised to initiate integrated care networks and are usually not considered as competitors by service providers [59,60]. To make a difference, they should be mandated with sufficient powers to draw up rigid guidelines for change. At the same time, they depend on the approval and participation of provider organisations in the field, which require inclusive processes of problem setting, direction setting and re-structuring [61]. Studies find that they need to strike a balance between facilitating cooperation among service providers and using the “shadow of hierarchy” [62].

At the *organisation level*, integrated care networks rely on the presence of motivated top managers with a vision of how their respective organisations gain from integrating care [8,44,103,104]. Since networks require time to develop, top managers with a longer tenure are more likely to develop formal and informal relationships with other organisational leaders required for advancing care integration [66].

### Leadership practices

A few studies analyse leadership media and leadership activities in their dynamic interplay. To consider these studies, a third category called “leadership practices” was developed, a term signifying a recursive relationship between social structure and action [31]. At the *network level*, a number of authors theorise network-based care integration as a recursive relationship between leadership media and actors’ responses to them. For instance, a recent study by Embuldeniya et al. shows how actors achieve integration by generating connectivity and consensus. These practices are situated in histories of existing cultures of clinician engagement and established partnerships. The study emphasises the recursive relationship between leadership media and activities, arguing that the identified practices are “contextually and temporally contingent, with the capacity to produce new contexts, which in turn generate new sets of mechanisms” [78, p. 783]. Other studies contribute to this perspective by showing how networks are formed and sustained through practices of co-creating [79], planning [80], sharing information [85], managing performance [81,82] and learning [83,84]. They find that leadership practices involve multiple distributed actors pooling resources and expertise [13,86,87].

A representative of practice-oriented research at the *policy level* is Tsasis et al., who analyse the formation and development of fourteen government-mandated integrated care networks in Canada [58]. They note that integration is challenged by a complex context including weak inter-organisational ties, financial dis-incentives and a bureaucratic command-and-control environment. Over time, distributed actors adjust this context through ongoing interactions, enacting practices like promoting system awareness, building relationships and sharing information. The authors point out that these practices constitute an evolving learning process rather than a series of programmatic steps.

At the *organisation level*, Patru et al. study the practices of boundary spanners in the formation and implementation of a Dutch healthcare network [101]. They show that by acting multilaterally, i.e. both within and across organisations over time, boundary spanners generate virtuous cycles in the development of network structures. Gurewich et al. examine how changes in hospital ownership structures affect hospitals’ pre-existing network ties with non-acute care providers [102]. They find that the effects of ownership transitions on the network are not linear but depend on the responses of actors at the network and policy level. These reactions are critical to determining how changes in hospitals’ ownership structures affect care for vulnerable populations.

### Leadership outcomes

This section sheds light on outcomes, analysing what leadership actually makes happen. Most studies target the *network level*, analysing how leadership affects network structures and the coordination of network activities. Regarding network structures, they explore how leadership impacts the formation of networks [8,44,47,58,61,68,69,71,82,90,100,101], the number and strength of network ties [51,56,63,64,67,77,78], network density [51,52], network centrality [9,51], network trust [70,72,75], network consensus [78] and network identity [84]. Regarding activity coordination, they examine how leadership affects patient and client referrals [7,10,48,50,52,53,59,60,62,65,66,79,89,91,98,102], care planning [7,45,48,54,55,57,80,93,95,96,97,102,104], information sharing [48,49,52,57,65,103], resource exchanges [7,52,99,102] and the alignment of care practices via protocols, pathways and evidence-based decision-making [13,46,73,87,88,92]. A few other studies focus on the *policy level* including citizens, patients and payers, showing how leadership influences access to care [11,12,42,56,66,74], service quality [13,41,42,43,66,74,83,85,86], efficiency [12,14,42,74,83] and care outcomes [12,56,76,83,86]. Relatively little is known how leadership relates to the *organisation level* of analysis. A few exceptions show how leadership enhances caregiver satisfaction [41,86] and the ability of providers to participate in a network [81].

## Discussion and suggestions for future research

Reviewing these findings, this section highlights gaps in the literature demanding attention in the future (see Table 2 for a summary). Addressing these gaps may help improve our currently limited understanding of leadership in integrated care networks [15].

**Table 2:** Opportunities for future research on leadership in integrated care networks.

Avenues for future research on leadership in integrated care networks		

Leadership mechanisms	1.	The field focuses on important leadership media but is rather silent on how actors implement and change these media. Future research could explore how actors proceed to create and re-create leadership media enabling and/or constraining the emergence and development of integrated care networks.
Levels of analysis	2.	The field tends to focus on the network level of analysis. Future research could examine in more depth how leadership happens at the neighbouring policy and organisation levels of analysis and how it affects and is affected by the network level.
Cross-cutting research themes	3.	Future research could explore how actors cope with inherent tensions, assuming that persistent contradictions between opposite elements are both media and consequences of leadership in integrated care networks.
	4.	Starting from a focus on integrated care, organisation and social network theory, the field could expand its theoretical base, including recent advances in leadership theory.
	5.	The majority of work focuses on outcomes at the network level. Future research could explore how leadership is related not only to the network itself, but also to patients’ experiences of care, population health outcomes, per capita spending and caregivers’ satisfaction, and how these outcomes enable and constrain subsequent leadership practice.

First, the findings indicate that the field tends to *focus on leadership media (58 studies)*. Important media include public governance structures, government legislation, funding, network governance, trust, and network members’ work traditions and practices. This work is crucial to our understanding of how these structures and processes enable and constrain integrated care networks. At the same time, it is rather silent on how actors implement and change these media. For example, observing how persistent organisational work routines constrain service integration, Glendinning suggests relinquishing traditional professional domains without explicating how this happens in practice [48]. Similarly, Retrum et al. find that favourable network outcomes depend on higher network density without elucidating how actors increase the number of network connections [75]. Future research could analyse how actors proceed to create and re-create these leadership media and explore required skills and competencies, starting from the valuable insights previous work has contributed to this area.

Second, the review perhaps unsurprisingly shows that the field tends to *focus on the network level of analysis (50 studies)*. Since networks are situated in a wider policy and organisational context, future research could usefully provide a fuller picture by exploring how leadership happens at these two levels and how it is related to the network level.

At the *policy level*, several studies emphasise that integrated care networks are mediated by in part conflicting public governance structures, government legislation, funding and performance control structures. They also point to the pivotal role of governments and health departments in forming integrated care networks as lead agencies. What remains less clear, however, is how these actors assume their role. As a rare exception, Voets et al. examine the role of governments in building an integrated youth care network in Belgium [62]. They find that, depending on the time and issue at hand, governments need to strike a balance between relying on autonomous interactions among network members and intervening hierarchically. Moreover, they show that “the government” is not a unity but involves a broad range of political and administrative actors whose activities are misaligned and need to be coordinated. This research raises important follow-up questions about policy actors’ identities, activities and practices. Further work is needed to disentangle the identities of influential policy actors, including, for example, politicians, political parties, government agencies or patient representatives. In addition, further research should be undertaken to better understand these actors’ activities and practices. For example, it would be important to know how they stabilise and change public governance structures, enact new legislation, provide funding and deal with providers to form networks and control their performance.

So far, comparatively little research has been carried out on linkages between the network and *organisation level*. The studies that do exist demonstrate that integrated care networks are enabled and constrained by network members’ priorities, resources, traditional work practices and available expertise. They also show that networks are affected by motivations and practices of network members’ senior leaders and boundary spanners. This paucity of research leaves ample room for progress. For example, further work is needed to establish how and why organisations decide to engage in integrated care networks in the course of their strategy process and how, in turn, collaboration feeds back on internal strategic considerations. Future research should also expand on the role of boundary spanners, who occupy a central position in bridging the network with member organisations. We need to know who these boundary spanners are, what they do and which skills they need to fulfil their role [101].

Third, the findings of the literature review suggest that integrated care networks are social systems imbued with *manifold tensions* [23,27,28,29]. To name but a few, they show that networks are persistently torn between market- and state-oriented public governance structures [9], collaboration and competition [42], collaboration and performance control [48,55,62], trust and capacity imbalances among network members [48,70], competing priorities within network members [43] and equifinal solutions to network governance questions [11]. In addition, the findings show that causalities are non-linear and contingent. Depending on the situation, networks are supported *or* constrained by pro-competitive policy reforms [6,41,42,43,44], government legislation [7,44,46,47], financial incentives [11,50,51,52] and intra-organisational changes like ownership transitions [102]. These findings correspond to wider network research, which argues that these tensions cannot be resolved and constitute a core challenge of network leadership [23]. Further empirical investigations are needed to explore how actors cope with this challenge, assuming that persistent tensions are both media and consequences of leading in integrated care networks.

Fourth, to increase its heuristic potential, the field could *expand its theoretical base*, which focuses on integrated care, organisation and social network theory. An obvious candidate that has received relatively scant attention is leadership theory, which has made progress e.g. in the areas of distributed and complexity leadership theory. Distributed leadership theory shifts the focus of analysis from the traits of individual leaders to the dynamics of “conjoint action” involving a variety of people, levels and organisations [107,108]. Buchanan et al., for instance, describe how the implementation of a UK cancer network was accomplished by “a large and shifting cast of formal and informal change agents in the absence of management plans, roles, and structures” [13, p. 1067]. Chreim et al. similarly show that the ability to influence the formation of integrated care networks is dispersed across multiple actors, with no single agent having full authority, resources or expertise to lead the change [86]. These findings raise interesting new questions about the extent to which distributed leadership is organised. While some researchers recommend dispensing with dedicated network coordinators to encourage fluid and migratory responsibilities [13], others argue that these coordinators play a key role in managing the process and building trust and relationships across organisations [86]. Moreover, complexity leadership theory builds on concepts from complexity science. Similar to distributed leadership theory, it assumes that networks are not designed through central control, but emerge from formal and informal combinations of multiple individual and situated actions [109]. As one of the few studies adopting this perspective, Tsasis et al. frame integrated care networks as complex adaptive systems [58]. They argue that building these systems requires leadership practices supporting relationship building and information sharing across professional and organisational boundaries. Future research could build on these insights, using recent advances in leadership theory to model how integrated care networks function and evolve.

A final avenue for future research concerns the *outcomes of leadership* in integrated care networks. The majority of work focuses on outcomes at the network level, including the coordination of activities and network structural variables like the number and strength of network ties. While these outcomes are important, future research should investigate the degree to which leadership affects not only the network itself, but also indicators at the policy and organisation level, in particular patients’ experience of care, population health outcomes, per capita costs of care provision and caregivers’ satisfaction [2].

This analysis has important practical implications. On the one hand, it provides practitioners with a conceptual map for navigating the different levels, media, practices and activities that need to be considered when exerting influence to create, develop and sustain integrated care networks. It summarises latest evidence from around the world practitioners can use to reflect on and improve their own leadership practice. On the other hand, it alerts practitioners to manifold tensions constituting leadership in integrated care networks. Torn by several contradictions between opposite elements across levels, leadership appears to be less orderly than perhaps expected. While these tensions interrupt routine, raise ambiguity and may lead to conflict, they are also important sources of change, providing practitioners with occasions for “reflexive structuration” [23]. Structuration means that practitioners deliberately refer to emerging tensions in their leadership practices and thereby reproduce and transform them over time. Reflexive means that practitioners accept tensions as the basic condition of their work and take precautions that the intended structuring works, for example by remaining alert to contradictory structures and processes, by exploring synergies between competing demands, by reframing tensions, and replacing either-or-assumptions with both-and-alternatives [29]. At the same time, unintended side effects remain to be expected, which constructs an evolving contradictory context for subsequent action and turns the handling of tensions into a persistent challenge of leading in integrated care networks.

## Concluding remarks

Around the globe, policy makers and service providers view collaboration in inter-organisational networks as a promising approach for improving health systems [1,2,4]. Against this background, this article set out to review the current state of knowledge about leadership in integrated care networks, a previously neglected topic in the literature [15]. Studies over the past decades have provided important information on the structures and processes making things happen in integrated care networks, with a particular focus on leadership and outcomes at the network level of analysis. Moreover, they have shed light on multiple tensions challenging leadership in integrated care networks, often drawing on integrated care, organisation and social network theory. Building on these valuable insights, further work could develop a deeper understanding of leadership activities, practices and outcomes across different levels, including the policy and organisation levels of analysis. In doing so, it could broaden its theoretical foundations, leveraging, for instance, recent advances in leadership theory.

Of course, like other reviews, this paper has several limitations, including its particular definitions of integrated care, networks and leadership and a rather narrow focus on peer-reviewed articles in English-language journals with high impact factors. These decisions restrict the scope of evidence reported in this review at the expense of relevant research published in other journals, monographs, edited books and languages. At the same time, they ensure a focus on state-of-the art and quality-controlled studies in the field.

Overall, this paper contributes to the literature by providing a comprehensive assessment of leadership in integrated care networks. It systematises the debate and outlines avenues for future research in this important and previously neglected sub-field of integrated care theory.

## Additional Files

The additional files for this article can be found as follows:

10.5334/ijic.5420.s1 Appendix 1. Matched search terms.

10.5334/ijic.5420.s2 Appendix 2. Studies included in the review (in alphabetical order).

10.5334/ijic.5420.s3 Appendix 2. Studies included in the review (in alphabetical order).
