# Supplementary material for: Leadership in Integrated Care Networks: A Literature Review and Opportunities for Future Research
Source: Int J Integr Care. 2020 Aug 11;20(3):6. doi: 10.5334/ijic.5420 (PMC7427680; doi:10.5334/ijic.5420)
Supplement: Appendix 1. — Matched search terms. [file ijic-20-3-5420-s1.pdf]

Appendix 1. Matched search terms

	Inter-organisational networks and synonyms	Leadership and synonyms	Integrated care and synonyms	Focus on inter-organisational networks
Search terms	network* or consorti* or cluster* or alliance* or collabor* or cooperat* or partnership*	control* or govern* or lead* or manag* or maneuv* or orchestrat*	integrated care or care coordinat* or shared care or coordinated care or co-ordinated care or comprehensive care or seamless care or transmural care or population health or accountable care	inter-organisational or interorganisational or inter-organizational or interorganizational or inter-sectoral or intersectoral
Application to	Title, abstract, keywords	Title, abstract, keywords	Title, abstract, keywords	Full text
