# Supplementary material for: Leadership in Integrated Care Networks: A Literature Review and Opportunities for Future Research
Source: Int J Integr Care. 2020 Aug 11;20(3):6. doi: 10.5334/ijic.5420 (PMC7427680; doi:10.5334/ijic.5420)
Supplement: Appendix 2. — Studies included in the review (in alphabetical order). [file ijic-20-3-5420-s2.pdf]

Appendix 2. Studies included in the review (in alphabetical order)

No.	Author	Year	Country	Journal	Theory	Method	Sample	Empirical context	Coded key findings
1	Adjerid et al.	2018	US	Information Systems Research	Transaction cost	quan	73	Data exchange	Spending reductions occur disproportionately in healthcare markets where providers have financial incentives to use a health information exchange.
2	Alexander et al.	2001	US	Medical Care	Organisation	quan	14	Primary and secondary care	Physicians in groups with more valued practice service linkages display higher alignment with health systems. Centralised administrative control lowers physician-system alignment.
3	Alidina et al.	2016	US	Health Care Management Review	Organisation	mixed	13	Primary care	Use of four coordination mechanisms (routines, information connectivity, boundary spanners and communication mechanisms) can help to improve care coordination in medical neighbourhoods.
4	Baum et al.	2016	Australia	Social Science & Medicine	Comprehensive primary health care (PHC)	qual	7	Primary care	Neo-liberal health sector reform reduces ability of primary health care services to implement comprehensive primary health care and inter-sectoral collaboration.
5	Bazzoli et al.	1998	US	Social Science & Medicine	Network	qual	6	Trauma care	Successful leaders spend substantial time and energy creating trust and shared understanding of values.
6	Bistaraki et al.	2019	UK	Public Health	Mass gatherings	qual	1	Public health	Nurturing inter-organisational linkages and creating shared understanding help create effective partnerships in mass gatherings.
7	Bode and Firbank	2009	Germany, UK, Canada	Policy Studies Journal	Governance	qual	3	Home care	Rather than providing for mutual adjustment, home-care networks tend to produce tensions and outcome problems because of the “biased” interplay between various steering rationales and different meta-governance regimes.

8	Breton et al.	2013	Canada	BMC Health Services Research	Integrated care	quan	23	Primary and secondary care	Local health network (LHN) reforms reduce collaborations outside new LHN areas and improve collaborations among healthcare organisations within new LHN areas.
9	Buchanan et al.	2007	UK	Human Relations	Leadership	qual	1	Cancer	Change roles in improving inter-organisational collaboration are distributed widely, with responsibilities migrating among a large informal cast of characters.
10	Bunger and Gillespie	2014	US	Health Care Management Review	Network	quan	1	Child and youth care	Cliques provide complementary services to similar client populations. Trust within cliques is higher. Members of cliques perceive greater efficiency and greater access to care and service quality.
11	Carstens et al.	2009	US	Journal of Behavioral Health Services & Research	Social ecology	qual	13	Child and youth care	Several factors affect the adoption decision, including the presence of entrepreneurial leadership.
12	Carter et al.	2014	UK	Implementation Science	Collective action	qual	1	Stroke	Collaboration conflicts with competition and variations in intra-organisational support.
13	Chang et al.	2017	Taiwan	International Journal of Medical Informatics	Social capital Transaction cost	quan	1	Data exchange	As parties enter into long-term contracts of cooperation and invest in relation specific assets, they develop greater trust in each other and further enhance each other's performance.
14	Chreim et al.	2010	Canada	Health Care Management Review	Leadership	qual	1	Primary care	The ability to initiate, influence and implement change is dispersed across the system.
15	Cristofoli and Markovic	2016	Switzerland	Public Administration	Network	qual	12	Home care	In resource-munificent contexts, various combinations of network structure and coordination mechanisms can lead to high network performance.

16	Davis et al.	2012	US	Psychiatric Rehabilitation Journal	Social network	mixed	1	Mental health	Grants provide funding for direct service provision. One of the least expensive ways to improve systems is to increase communication across organisations, ensuring exchange of critical information about clients as they move from one type of service to the next, and to raise awareness of their unique needs.
17	Dinesen et al.	2011	Denmark	International Journal of Integrated Care	Network Innovation	qual	1	COPD	Obstacles are identified in the network context; these obstacles include the mind-set of the healthcare professionals, inter-professional relations, views of technology as a tool and competing visions for the goals of tele-rehabilitation.
18	Embuldeniya et al.	2018	Canada	Milbank Quarterly	Practice	qual	6	Primary and secondary care	Integration is mediated by chosen program structures and generated by creating connectivity and consensus.
19	Fleishman et al.	1992	US	Social Service Review	Network	qual	9	HIV	Inter-organisational dynamics are most positive when health departments are lead agencies. Having health departments as lead agencies has the advantage of stability, legitimacy, and administrative capability to monitor grants and contracts.
20	Fleury et al.	2002	Canada	International Journal of Health Planning and Management	Network	qual	1	Mental health	To develop an integrated network, an organisation must be mandated with extensive powers to draw up rigid guidelines for the system restructuration. However, a reform can really be implemented only with the approval and genuine participation of the actors directly involved in the field. This stresses the importance of the ongoing process of forming networks, i.e. problem-setting, direction-setting, structuring.
21	Foster-Fishman et al.	2001	US	American Journal of Community Psychology	Network	mixed	2	Public health	Organisations involved in coordinating councils and inter-agency teams are more likely to be included in client, information, and resource exchanges.

22	Fuller et al.	2015	Australia	BMJ Open	Network Complexity	mixed	1	Mental health	Using facilitated reflection, health and social care organisations are able to see their linkages and identify as a network, thereby identifying the need for a network administration organisation as the network manager with credibility and a mandate across health and social care sectors.
23	Gamm and Benson	1998	US	Journal of Health Politics, Policy and Law	Organisation	qual	3	Hospital networks	The presence of strong leadership and commitment is part of the reason for some of the successes in two regions. The lack of strong leadership may help to account for a weakness in another effort.
24	Glendinning	2003	UK	Health Policy	Integrated care	mixed	104	Primary and secondary care	Major barriers to integration remain: professional domains and identities, status differentials, the wider policy environment, vertical relationships with national government.
25	Goldman et al.	2010	Canada	Journal of Interprofessional Care	Knowledge	qual	14	Family care	Leadership at various levels is key to successful local implementation.
26	Greenhalgh et al.	2016	Australia	Milbank Quarterly	Knowledge	qual	1	Primary care	Principles of successful co-creation include a systems perspective, a creative approach to research focused on improving human experience and careful attention to governance and process.
27	Grimshaw et al.	2010	UK	Human Resource Management Journal	HR	qual	2	Primary and secondary care	Differences in organisational goals and HRM approaches in hospital networks are an obstacle to long-term integration.
28	Grusky	1995	US	Administration and Policy in Mental Health	Resource dependence Contingency Leadership	quan	8	Mental health	Long tenure of network members' directors, a powerful lead agency and system integration enhance the effectiveness of county mental health systems.

29	Guerrero et al.	2014	US	American Journal of Public Health	Integrated care	quan	104	Addiction health	Components of program capacity such as public funding and organisational readiness for change play a significant role in the coordination of mental health and public health in community-based addiction health services programs.
30	Gurewich et al.	2003	US	Milbank Quarterly	Network	qual	5	Community care	The actions of community organisations and public policy can be critical to determining how changes in a hospital's ownership affect care for vulnerable populations.
31	Hermens et al.	2019	Netherlands	Sport Management Review	Integrated care	quan	32	Sport-for-health	Actors in sport-for-health partnerships should pay particular attention to communication structure, building on capacities, visibility, and task management.
32	Hjelmar et al.	2011	Denmark	International Journal of Integrated Care	Resource dependence Network Motivation	qual	1	Elderly care	Care providers' motivational factors need to be addressed to fully exploit the potential health benefits of cross-sectoral programs.
33	Jain et al.	2016	US	Health Education & Behavior	Social network	quan	4	HIV	Program funding may increase some collaboration types among organisations serving people living with HIV.
34	Janssen et al.	2015	Netherlands	Health Policy	Organisation	qual	1	Elderly care	Signs of empowering organisational features are mutual trust and clear working routines.
35	Javanparast et al.	2019	Australia	Australian and New Zealand Journal of Public Health	Institutional	mixed	96	Public health	Organisational capacity and resources, supportive governance and public health legislation mandating a role for local governments are critical to collaborative planning.
36	Johnson et al.	2003	UK	Journal of Interprofessional Care	Collective action	qual	4	Community care	Where goals of providers are congruent, collaboration occurs more smoothly.
37	Kominis and Dudau	2012	UK	Management Accounting Research	Control	qual	1	Child and youth care	Attempting to facilitate the interaction of different actors, government complements diagnostic with interactive control systems.

38	Korst et al.	2011	US	International Journal of Medical Informatics	Innovation	quan	1	Data exchange	The presence of leaders' vision for how the collaborative advances the hospital's strategic goals is a key success factor.
39	Kramer et al.	2012	Germany	BMC Family Practice	Planned behaviour	qual	1	Heart care	Key factors affecting the implementation of an inter-sectoral care pathway are professionals' previous behaviour and (conflicting) national program guidelines.
40	Kurunmäki and Miller	2011	UK	Management Accounting Research	Governmentality	qual	5	Learning disabilities<br>Child care<br>Elderly care	Cooperation can be enhanced through the creation of settings in which information can be exchanged, formally separate administrative groups can meet, and instruments can be devised that provide visibility to the norms and expectations of different parties. This allows the assessment of instruments, policies and time-lines, together with negotiation over their alignment.
41	La Rocca and Hoholm	2017	Norway	BMC Health Services Research	Integrated care	qual	1	Primary and secondary care	ICT-based communication is perceived to facilitate information exchange between primary and secondary care, thus positively affecting coordination. However, the economic incentive scheme appears to have the opposite effect by creating tensions between organisations and accentuating power asymmetry in favour of secondary care.
42	Larsson et al.	2019	Sweden	International Journal of Health Planning and Management	Network	quan	20	People with complex needs	Inability to collaborate in patient care planning may be due to shortcomings in terms of trust between caregivers in the health care organisation at a national level.
43	Lezwijn et al.	2014	Netherlands	Health Promotion International	Planning	qual	3	Elderly care	In practice, several forms of planning approaches are used, depending on the degree of complexity and dynamics of the context, the phase of the health promotion programme, and the time available.

44	Li et al.	2018	UK	Health Policy	Network	qual	3	Primary and secondary care	Trust among integrated care service provider networks is influenced by recognition and knowledge asymmetries, capacity and financial imbalances, organisational differences in management, culture and attitudes toward change.
45	Lin et al.	2012	Taiwan	Journal of Medical Systems	Innovation	quan	1	Data exchange	Environmental pressure (competition, government mandate) and top management attitude are critical factors influencing hospitals' intention on whether to adopt a health information exchange standard.
46	Luke et al.	2010	US	American Journal of Public Health	Social network	quan	5	Public health	Lead agencies are more likely to be involved in partnerships. Partners are more likely to work together if they are physically close.
47	Lukeman et al.	2019	Canada	BMC Health Services Research	Social network	mixed	1	Breast-feeding	Relationships and trust are connected to knowledge exchange.
48	Lyngso et al.	2016	Denmark	International Journal of Integrated Care	Integrated care	qual	1	COPD	Committed leadership with clear communication processes and front-line staff members who are willing to take responsibility for communication are crucial when bringing different cultures together.
49	Marks et al.	2011	UK	Journal of Health Services Research & Policy	Governance	mixed	10	Public health	Performance management regimes and incentives are described as geared towards the success of single organisations rather than partnerships, with tensions between national targets and local priorities.

50	Martin et al.	2009	UK	Journal of Public Administration Research and Theory	Leadership	qual	2	Cancer	Leadership needs to be dispersed as well as distributed to engage diverse, powerful stakeholder groups, and this implies a variety of leaders as well as leadership styles. The inter-organisational network itself is ineffective as a means of achieving change, but it does provide the space and the media for leaders to effect change.
51	Meijboom et al.	2004	Netherlands	Health Policy	Organisation	conc	n/a	n/a	The notion of inter-clan provides the economic rationale for health networks. An essential condition in favour of the introduction of the inter-clan is the transfer of specialised expertise through mutual professional trust.
52	Meijboom et al.	2010	Netherlands	International Journal of Health Planning and Management	Supply chain	conc	n/a	n/a	Structures applying cross-functional and cross-organisational teams can help eliminate integration problems related to unwillingness to change.
53	Minkman et al.	2009	Netherlands	Health and Social Care in the Community	Integrated care	qual	8	Dementia care	Factors for success concern investment in a strong provider network. Failure factors are distrust, competition for delivering care and inadequate program funding.
54	Morgan et al.	2019	New Zealand	Health and Social Care in the Community	Integrated care	qual	2	Child and youth care	Establishing and maintaining trusting inter-personal relationships with individual staff is key to successfully negotiating agency differences.
55	Nuti et al.	2018	Italy	Management Decision	Control	qual	13	Secondary care	Re-framing performance management systems supports the alignment of professionals' and organisations' goals and behaviours.
56	Owusu et al.	2013	Ghana	International Journal of Integrated Care	Integrated care	mixed	2	Malaria control	Even in a setting characterised by high levels of social capital, institutions are less likely to function as a coordinated system if there are differences in organisational agenda, expectations and lack of trust.

57	Page	2003	US	Health Care Management Review	Network	conc	n/a	n/a	Over time, continuous improvement practices may help build strong collaborative cultures that reinforce their own success, if participants implement them incrementally and adapt them based on experience.
58	Patru et al.	2015	Netherlands	Medical Care Research and Review	Network	qual	1	Primary and secondary care	By acting multilaterally, boundary spanners generate virtuous cycles in the development of the network.
59	Pucher et al.	2015	Netherlands	BMC Public Health	DISC	mixed	5	Public health	Five main management styles to improve inter-sectoral collaboration in the initial stage: 1) facilitating active involvement of relevant parties; (2) informing collaborating parties; (3) controlling and (4) supporting their task accomplishment; and (5) coordinating the collaborative processes.
60	Retrum et al.	2013	US	Health Education & Behavior	Social network	quan	99	Public health	The structural signature that had the most significant relationship to outcomes was density, with higher density indicating more positive outcomes. Also significant was the finding that more breadth creates new challenges such as difficulty in reaching consensus and creating ties with other members.
61	Rosenheck et al.	2001	US	Health Services Research	Social capital	quan	18	Homeless care	Social capital is associated with greater service systems integration.
62	Scheele and Vrangbaek	2016	Denmark	International Journal of Integrated Care	Integrated care	qual	1	Community care	Co-location does not function as a driver for cross-sectoral collaboration in a health centre. Cross-sectoral collaboration is hampered by the general practitioners' work routines and professional identity and by a lack of clarity concerning the content of collaboration with regard to economic and professional incentives.

63	Short et al.	2015	Australia	Health Promotion International	Network Organisation	qual	1	Community care	Credible leadership facilitates inter-professional collaboration and network participation by promoting the network to members, the broader health and social sectors, including government policy offers; by maintaining motivation and engaging network members; by providing focus and structure of the network; by facilitating understanding of each other's perspectives.
64	Spear	2014	US	Drug and Alcohol Dependence	Social network	quan	32	Alcohol dependence	Network efficiency (proportion of ties within a network that are non-redundant) is associated with lower odds of readmission.
65	Timpka et al.	2007	Sweden	Journal of Biomedical Informatics	Actor Network	qual	1	Mental health	Three interventions must be regarded when configuring an information infrastructure for mental health services: (1) an integrated service policy defined by the national government, (2) a common legal framework allowing sharing of high-level client data, (3) and commissioned support for local inter-agency workspaces.
66	Tsasis et al.	2012	Canada	International Journal of Integrated Care	Complexity	qual	1	Community care	Health systems integration requires policies and management practices that support relationship building and information-sharing across organisational and professional boundaries, and that recognise change as an evolving learning process rather than a series of programmatic steps.
67	Tung et al.	2018	US	Health Services Research	Integrated care	qual	1	Diabetes	Among diverse motivations across organisation types, stakeholders describe collaboration as an opportunity for: financial support, brand enhancement, access to specialised skills or knowledge, professional networking, and health care system involvement in community-based efforts.

68	Van Haute et al.	2018	Belgium	Child & Family Social Work	Panopticon and pastoral power	qual	3	Child poverty	It is undesirable to formally protocol and pre-structure inter-organisational information exchange practices. Rather than fixed and standardised regulations, a more pedagogical, dialogical, and transparent negotiation between network partners and families can be suggested.
69	Vendetti et al.	2017	US	Addiction	Screening and brief intervention (SBI)	mixed	7	Substance abuse	Interviewees often mention a specific ‘champion’, a charismatic leader, who facilitates implementation by delivering strong, consistent messages regarding program importance, and by encouraging communication among key stakeholders.
70	Voets et al.	2015	Belgium	Public Management Review	Governance	qual	1	Child and youth care	Framing, designing, managing and participating in a collaborative governance regime requires meta-governors to know when to allow for autonomy and dialogue, and when to use the ‘shadow of hierarchy’.
71	Westra et al.	2017	Netherlands	Health Policy	Strategic management	conc	n/a	n/a	Cooperation among providers is influenced by pro-competitive reforms, overlap in geographic and product markets and network outcomes.
72	Wiktorowicz et al.	2010	Canada	International Journal of Integrated Care	Network	qual	10	Mental health	Mental health networks adopt either a corporate structure, mutual adjustment or an alliance governance model. A corporate structure, in which a central authority, e.g. a regional health authority, develops written expectations, supported by regionalisation, offers the most direct means for local governance to attain inter-organisational collaboration.
73	Wright and Shuff	1995	US	Social Networks	Social network	quan	19	HIV	Positive effects for being near a care coordination site and for being a member of a cohesive network of mental health centres were observed.
